# Anti‐Aging Effects of *Schisandrae chinensis* Fructus Extract: Improvement of Insulin Sensitivity and Muscle Function in Aged Mice

**DOI:** 10.1155/2019/5642149

**Published:** 2019-11-03

**Authors:** Hojung Choi, Eunhui Seo, Myeonghoon Yeon, Myung-Sunny Kim, Haeng Jeon Hur, Byung-Chul Oh, Hee-Sook Jun

**Affiliations:** ^1^College of Pharmacy and Gachon Institute of Pharmaceutical Sciences, Gachon University, Incheon 21936, Republic of Korea; ^2^Lee Gil Ya Cancer and Diabetes Institute, Gachon University, Incheon 21999, Republic of Korea; ^3^Division of Nutrition and Metabolism, Korea Food Research Institute, Wanju-gun, Jeollabuk-do, Republic of Korea; ^4^Department of Physiology, College of Medicine, Gachon University, Incheon 21999, Republic of Korea; ^5^Gachon Medical Research Institute, Gil Hospital, Incheon 21565, Republic of Korea

## Abstract

*Schisandrae chinensis* Fructus has a long history of medicinal use as a tonic, a sedative, and an antitussive drug. In this study, we investigated the beneficial effects of *Schisandrae chinensis* Fructus ethanol extract (SFe) on metabolism in an aged mouse model. Sixteen-month-old C57BL/6J mice were fed with a diet supplemented with SFe for 4 months. Insulin sensitivity was lower at 20 months of age than at 16 months of age; however, the decrease in insulin sensitivity was less in SFe-fed mice. SFe supplementation also appeared to improve glucose tolerance. Body weight gain was lower in SFe-fed mice than in mice fed the control diet. Body fat mass was lower and the lean mass was higher in SFe-fed mice. In addition, the grip strength was enhanced in SFe-fed mice. Histological analysis of the tibialis anterior muscle showed that the size of myofiber and slow-twitch red muscle was increased by SFe supplementation. The expression of proteins related to muscle protein synthesis such as phospho-Erk1 and phospho-S6K1 was increased by SFe supplementation. The mRNA expression of genes related to myogenesis and their encoded proteins such as MyoD, Myf5, MRF4, myogenin, and myosin heavy chain, was increased, whereas that of genes related to muscle degradation, such as atrogin-1, MuRF-1, and myostatin, were decreased relative to control mice. These results suggest that SFe supplementation might have beneficial effects for the improvement of insulin sensitivity and inhibition of muscle loss that occur with aging.

## 1. Introduction

Aging is defined as decline in the function of organisms over time and is likely caused by spontaneous loss of intracellular and intercellular functions [1, 2]. Therefore, aging is accompanied by declines in physical and cognitive functions; moreover, aging is the main risk factors for chronic diseases such as diabetes, obesity, neurodegenerative conditions, and cancer [3].

Age-induced muscle loss is the progressive loss of skeletal muscle mass and strength, which results in a decreased quality of life [4]. Although the exact mechanism is not known, many factors are responsible for the decreased muscle mass and strength in the elderly, such as increased insulin resistance, inflammation, hormonal alterations, perturbations in muscle metabolism, and decreased muscle proliferation [5]. Insulin resistance is known to lead to a reduction in muscle synthesis in elderly individuals. Furthermore, hyperglycemia is a risk factor for age-induced reduction of muscle mass and function [6]. In a human study, it was shown that increased muscle mass was related to improved insulin sensitivity [7].

Given the increases in life expectancy, increases in prevalence of age-related diseases are expected to present a global social and economic problem. Therefore, efficient methods for health intervention or health promotion should be developed to minimize the cost and enhance the quality of life for the aging population. Currently, there is considerable interest within the research field for the identification of natural products to combat age-related functional declines.

*Schisandrae chinensis* Fructus (SF) is widely used in traditional medicine as a therapy for asthma, night sweats, insomnia, dry cough, urinary disorders, involuntary ejaculation, poor memory, hyperacidity, chronic diarrhea, hepatitis, and diabetes in Korea, China, and Russia. SF contains many lignans, including schisandrin A, schisandrin B, schisandrin C, schisandrol A, and schisandrol B, and other impurities [8–10]. The known pharmacological effects of SF include antioxidative, antitumor, hepatoprotective, chondroprotective, antiseptic, anti-inflammatory, anti-atherosclerotic, and antidiabetic activities [11–15]. Recent studies have suggested that the administration of SF also has a beneficial effect on muscle metabolism and myogenic differentiation [16] and dexamethasone-induced muscle atrophy [17]. However, it is not known whether SF has a preventive effect on natural aging-associated insulin resistance and muscle loss. In this study, we investigated the effect of ethanol extract of SF on insulin resistance and muscle loss, which are subject to age-related functional decline, in aged mice.

## 2. Materials and Methods

### 2.1. Preparation of SF Ethanol Extract (SFe)

*Schisandra chinensis* grown in the Korean peninsula was used in the experiments. SFe was prepared in accordance with a previous reported procedure [18] with a slight modification. Briefly, dried SF was purchased from a local market (Seoul, Korea). One hundred grams of powdered SF was extracted three times in 900 ml of 70% ethanol by shaking for 24 h at 25°C. The precipitate was removed by centrifugation at 8,000 ×g for 30 min (Beckman, Brea, CA). Finally, the supernatant was lyophilized in a freeze dryer (Il Shin, Korea).

### 2.2. Supplementation with SFe

Twelve-month-old and 16-month-old-male C57BL/6J mice were purchased from the Korea Research Institute of Bioscience and Biotechnology (Daejeon, Korea). The animals were housed under specific-pathogen-free conditions and maintained under a 12 h/12 h light/dark cycle at the animal facility of Lee Gil Ya Cancer and Diabetes Institute (CACU, Gachon University, Incheon, Korea), in accordance with “Guidelines for Animal Users” (LCDI-2014-0006). After a 1-week adaptation period, the 16-month-old mice were divided into two groups with similar weight and blood glucose levels. Subsequently, one group was fed a normal diet (AIN-93G, Research Diet, Inc., New Brunswick, NJ, USA) and the other group was fed a normal diet supplemented with 0.1% SFe (customized by Research Diets) for 4 months. In addition, 12-month-old mice were fed with the normal diet for 4 months, without 0.1% SFe supplementation, until the mice reached 16 months of age. Body weight and food intake were measured at 2-week intervals.

### 2.3. Measurement of Body Fat Composition and Grip Strength

Fat and lean body mass were assessed using a ^1^H minispec system (Bruker BioSpin). An automated grip strength meter (Columbus Instruments, Columbus, OH) was used to measure the forelimb grip strength. The mean force in grams was determined by using a computerized electronic pull strain gauge that was fitted directly to the grasping ring. The maximal force obtained from three tests was used as the dependent measure.

### 2.4. Glucose Tolerance Tests

Mice were fasted overnight, and glucose (2 g/kg) was administered by intraperitoneal injection. Blood samples were obtained from the tail vein at 0, 30, 60, 90, and 120 min after glucose injection, and blood glucose levels were measured using a glucose analyzer (OneTouch® Ultra, Lifescan, Johnson & Johnson, Milpitas, CA, USA).

### 2.5. Insulin Tolerance Tests

Mice were fasted for 4 h, and insulin (0.75 U/kg body weight; Humilin; Lilly, Indianapolis, IN) was administrated by intraperitoneal injection. Blood samples were obtained from tail vein at 0, 30, 60, 90, and 120 min after glucose injection, and blood glucose levels were measured using a glucose analyzer (OneTouch® Ultra, Lifescan, Johnson and Johnson, Milpitas, CA, USA).

### 2.6. Histological Analysis of Tibialis Anterior (TA) Muscle

The muscle tissues were fixed in 10% neutral buffered formalin, embedded in paraffin, and sectioned. The sections were stained with hematoxylin and eosin (H&E) or periodic acid-Schiff (PAS) in accordance with the following procedure: briefly, slides were deparaffinized by incubation in xylene, hydrated by washes in a graded series of ethanol solutions (100%, 95%, 80%, and 70%), washed in distilled water, and then stained with H&E. For PAS staining, the tissue sections were incubated with periodic acid solution (Sigma-Aldrich, St. Louis, MO, USA) for 20 min followed by Schiff solution for 20 min.

### 2.7. Western Blotting

The tissues were lysed using a protein extraction kit (GE Healthcare, Piscataway, NJ, USA). Proteins samples (30 *μ*g) were separated by 10% SDS-PAGE and electrophoretically transferred to a polyvinylidene difluoride membrane. The membrane was blocked by incubation in 5% skim milk for 1 h and subsequently incubated with primary antibodies against phospho-Erk1/2 (#9101, Cell Signaling, USA), Erk1/2 (#9102, Cell Signaling), phospho-S6K (#9205, Cell Signaling), S6K (#9202, Cell Signaling), myogenin (MyoG) (sc-52903, Santa Cruz Biotechnology, USA), Myf5 (ab125301, Abcam), MyoD (sc-377460, Santa Cruz Biotechnology, USA), myosin heavy chain (MyHC) (sc-20641, Santa Cruz Biotechnology), muscle atrophy F-box/atrogin-1 (atrogin-1) (ab74023, Abcam, USA), muscle RING-finger protein-1 (MuRF-1) (ab172479, Abcam), myostatin (MSTN) (ab203076, Abcam), or GAPDH (sc-32233, Santa Cruz Biotechnology), which was used as a loading control. The membrane was washed three times with TBST (100 mM Tris pH 7.4, 150 mM NaCl, and 0.5% Tween-20) for 10 min and then incubated with horseradish peroxidase-conjugated goat anti-rabbit IgG (A120-101P, Bethyl, USA) or horseradish peroxidase-conjugated goat anti-mouse IgG (A90-116P, Bethyl, USA) secondary antibodies. The signals from the antibodies were detected by using a Fujifilm Luminescent Image Analyzer LAS4000 after the application of an ECL detection kit (Merck Millipore, Darmstadt, Germany). The experiment was performed using 5 or 10 samples from each group, as indicated in figure legends. The blots were quantified using ImageJ software (NIH, Bethesda, MD, USA).

### 2.8. Quantitative Real-Time-PCR (qRT-PCR) Analysis

Total RNA from TA muscle was prepared by using RNAiso reagent (Takara, Otsu, Japan) in accordance with the manufacturer's protocol. Reverse transcription of 2 *μ*g of total RNA was conducted by using the 1st strand cDNA synthesis kit (Takara, Otsu, Japan) in a reaction volume of 20 *μ*l. mRNA expression was analyzed by qRT-PCR using SYBR green reagent (Takara, Otsu, Japan); each experiment was performed using 5 or 10 samples from each group, as indicated in the figure legends. The sequences of primer pairs are listed in Table 1.

### 2.9. Statistical Analysis

The statistical significance of differences between groups was determined using one-way analysis of variance (ANOVA), followed by Fisher's LSD test computed in GraphPad Prism software. Significance was determined if *p* values were less than 0.05.

## 3. Results

### 3.1. Supplementation with SFe Improved Insulin Sensitivity and Glucose Tolerance of Aged Mice

**Figure 1:**
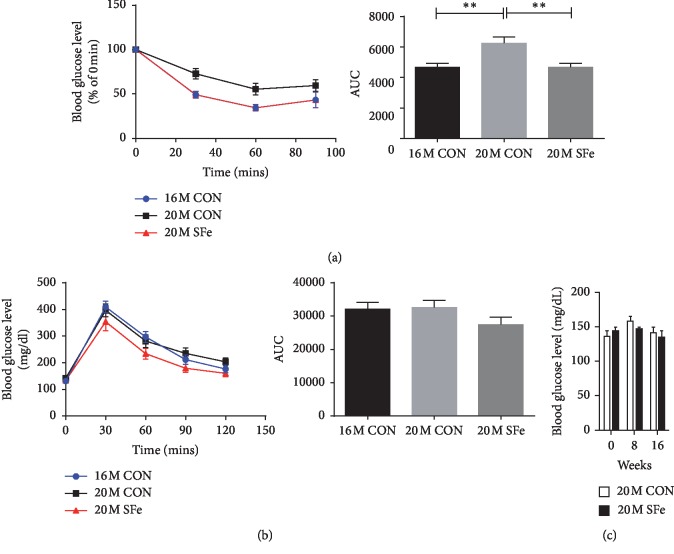
Supplementation with SFe improved the insulin sensitivity and glucose tolerance of aged mice. Sixteen-month-old male C57BL/6J mice were fed a diet of AIN-93G with or without SFe for 4 months. As a control, 12-month-old male C57BL/6J mice were fed a diet of AIN-93G for 4 months. Insulin tolerance tests (a) and glucose tolerance tests (b) were performed after 4 months (*n* = 14 per group). (c) Non‐fasting blood glucose level was measured at 0, 8, or 16 weeks after supplementation with SFe (*n* = 14 per group). The data are presented as the mean ± standard error (SE). ^*∗∗*^*p* < 0.01.

To evaluate the effect of SF ethanol extract (SFe) on metabolism in aging, 16-month-old mice were fed a normal diet (without SFe) until they reached 20 months of age (20M CON); subsequently, insulin tolerance tests (ITT) were performed. Insulin sensitivity was significantly lower in the 20M CON group than in the 16-month-old mice (16M CON) group. However, insulin sensitivity in the 20-month-old mice fed with a diet containing SFe (20M SFe) for 4 months was similar to that in the 16M CON group (Figure 1(a)). Glucose tolerance tests (GTT) showed that there was no difference between the 16M CON group and 20M CON group. Glucose tolerance appeared to be improved by supplementation with SFe, although the difference was not significant (Figure 1(b)). We also assessed the nonfasting blood glucose levels at 0, 8, or 16 weeks after supplementation with SFe (Figure 1(c)). Although a decreasing tendency in blood glucose levels was observed, no significant changes between the 20M CON and 20M SFe groups were detected (Figure 1(c)).

### 3.2. Supplementation with SFe Decreased Fat Mass and Increased Lean Mass of Aged Mice

**Figure 2:**
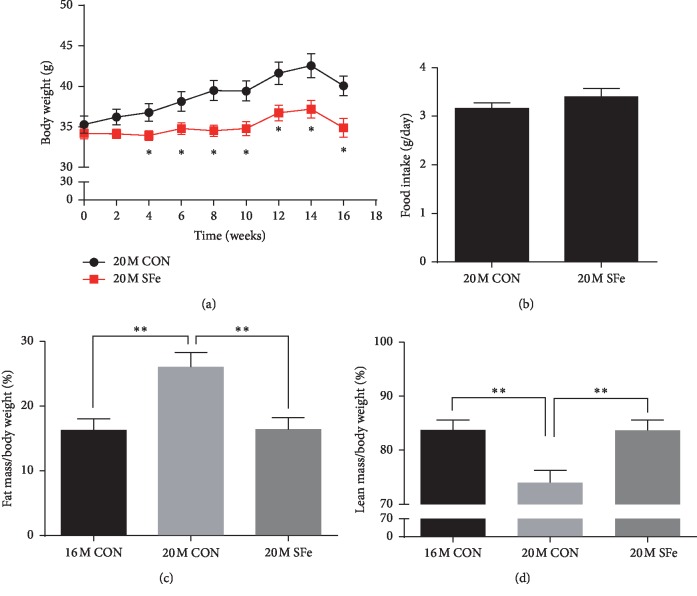
Supplementation with SFe decreased the fat mass but increased the lean mass in aged mice. Sixteen-month-old male C57BL/6J mice were fed a diet of AIN-93G with or without SFe for 4 months. (a) Body weight. (b) Food intake. (c) Body fat mass. (d) Body lean mass (*n* = 14 per group). The data are presented as the mean ± standard error (SE). ^*∗*^*p* < 0.05 and ^*∗∗*^*p* < 0.01.

Body weight was increased in mice fed with the control diet for 4 months (20M CON group), but this gain in body weight was inhibited in mice supplemented with SFe (20M SFe group; Figure 2(a)); however, the food intake between two groups was not different (Figure 2(b)). An analysis of the body composition using the ^1^H minispec system revealed that the fat mass was significantly higher in the 20M CON group than in the 16M CON group; however, this increase was not observed in the 20M SFe group. In contrast, the lean mass was lower in the 20M CON group than in the 16M CON group, but this decrease was restored in the 20M SFe group (Figures 2(c) and 2(d)). These data indicated that supplementation with SFe decreased fat mass and increased lean mass in aged mice.

### 3.3. Supplementation with SFe Enhanced the Muscle Function and Increased Myofiber Size of Aged Mice

**Figure 3:**
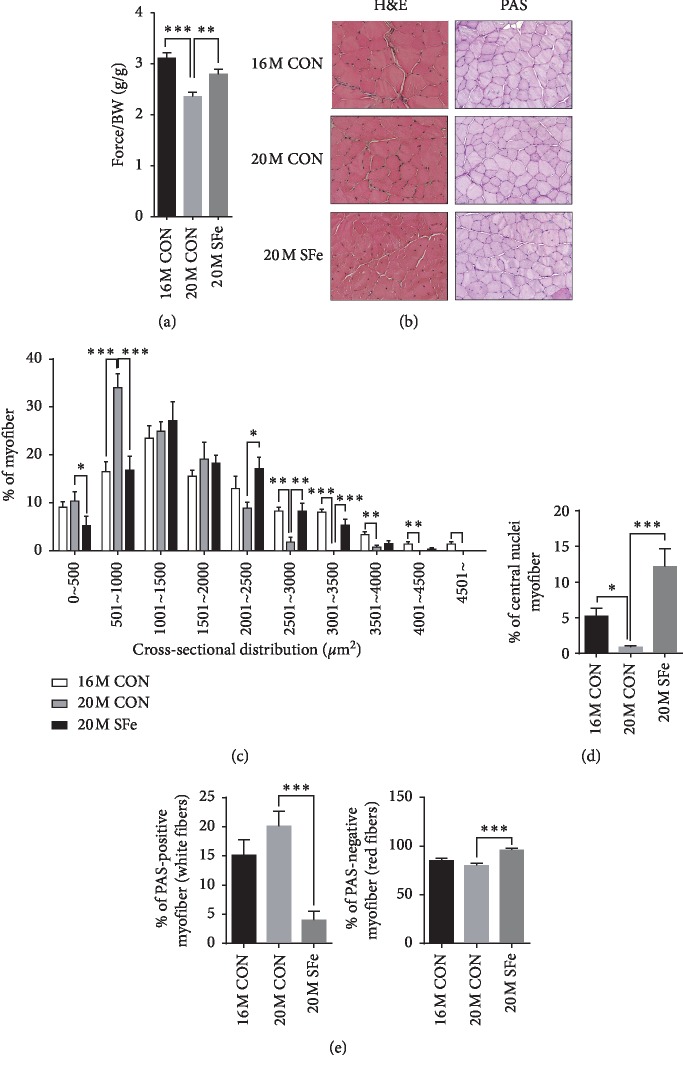
Supplementation with SFe enhanced the grip strength and increased myofiber size of aged mice. (a) Grip strength. The data are the mean ± standard error (SE) (*n* = 11 per group). ^*∗∗*^*p* < 0.01; ^*∗∗∗*^*p* < 0.001 (b) H&E and PAS staining of TA muscle sections. Representative images are shown (×200) (*n* = 3 per group). (c) The cross-sectional area (CSA) distribution of muscle fiber was measured using ImageJ program, and the average CSA are shown (*n* = 3 per group). (d) The percentage of myofiber with central nuclei in TA muscle (*n* = 3 per group). (e) PAS-stained positive and negative myofibers were counted using ImageJ program, and the data are expressed as a percentage of the number of total myofibers. Data are presented as the mean ± standard error (SE) (*n* = 3 per group). Three different areas per sample were analyzed. ^*∗*^*p* < 0.05; ^*∗∗*^*p* < 0.01; and ^*∗∗∗*^*p* < 0.001.

The decrease in body lean mass that occurs upon aging is accompanied by a decrease in muscle function [19]. As we found that SFe supplementation restored the lean mass, we evaluated the muscle function through the measurement of the grip strength, as described in Materials and Methods. The grip strength was weaker in the 20M CON group than in the 16M CON group and significantly stronger in the 20M SFe group than in the 20M CON group (Figure 3(a)). We then compared the myofiber area in the TA muscle by H&E staining. As shown in Figures 3(b) and 3(c), the cross-sectional area (CSA) decreased in the 20M CON group compared to the 16M CON group. The CSA significantly increased upon SFe supplementation. The number of multinucleated myofibers was higher in the 20M SFe group than in the 20M CON group (Figure 3(d)). To investigate the effects of SFe on different muscle types, TA muscle was stained with PAS. PAS can differentiate oxidative red (slow twitch) fibers from glycolytic white (fast twitch) fibers according to glycogen content [20–22]. The number of oxidative red fibers did not differ between the 20M and 16M CON groups but was significantly increased in the 20M SFe group compared with the 20M CON group (Figures 3(b) and 3(e)).

### 3.4. Supplementation with SFe Increased the Expression of Proteins Related to Muscle Synthesis in Aged Mice

**Figure 4:**
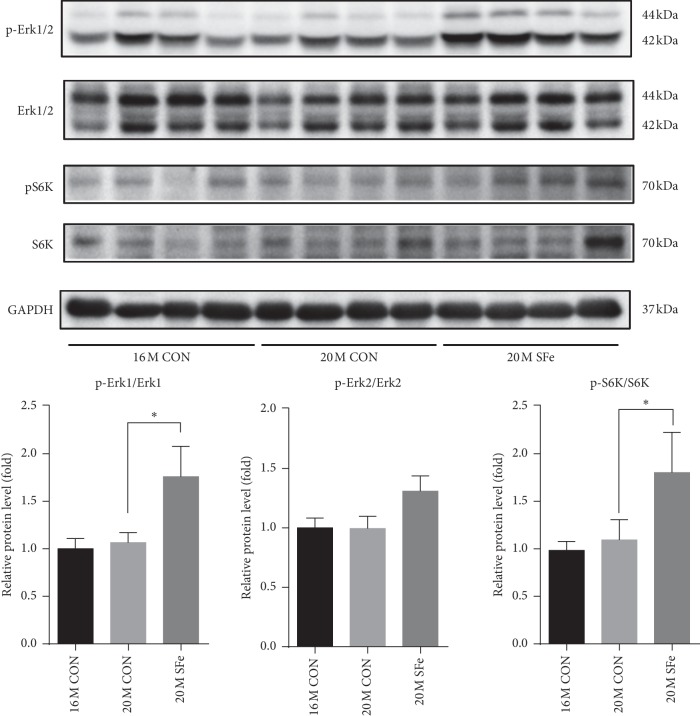
Supplementation with SFe increased muscle protein synthesis in aged mice. Phospho-Erk1/2, Erk1/2, phospho-S6K, and S6K protein expression in TA muscle were analyzed by western blotting. GAPDH expression was analyzed as an internal control. Representative images of the blots are shown in the upper panel; quantification of the blots was computed using ImageJ software and is shown in the lower panel. Data are presented as the mean ± standard deviation (SD) (*n* = 10 per group). ^*∗*^*p* < 0.05.

Since we observed that muscle strength and myofiber size were increased by dietary supplementation of SFe in 20-month-old mice, we investigated whether SFe supplementation increases muscle protein synthesis in TA muscle. Erk1/2 phosphorylation and S6K phosphorylation promote the differentiation and protein synthesis of myotube [23, 24]. We found that the phosphorylated Erk1/2 and S6K protein levels were increased by SFe supplementation in the 20M SFe group than in the 20M CON group (Figure 4). These data indicated that supplementation with SFe might result in an increase in muscle protein synthesis in aged mice.

### 3.5. Supplementation with SFe Increased Myosin Heavy Chain Expression in Aged Mice by Enhanced Myogenesis

**Figure 5:**
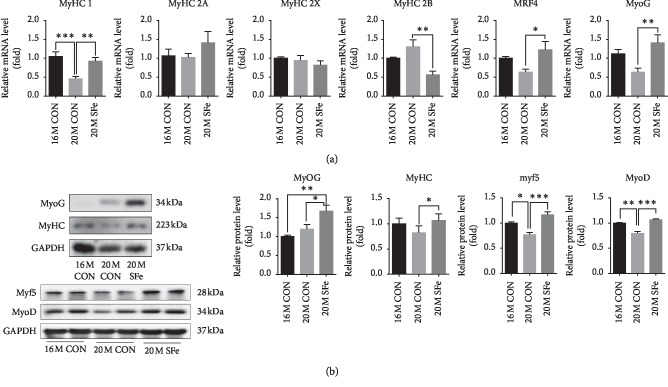
Supplementation with SFe increased muscle protein synthesis in aged mice. (a) The mRNA expression of MyHC-1, -2A, -2X, -2B, MRF4, and MyoG in TA muscle was analyzed by using qRT-PCR, with 18S rRNA used as an internal control. The values are expressed as the relative fold change as compared with the 16M CON group. (b) MyoG, MyHC, Myf5, and MyoD protein expression in TA muscle were analyzed by western blotting, with GAPDH expression analyzed as an internal control. Representative images of the blots are shown in the left panel; quantification of the blots was computed using ImageJ software and is shown in the right panel. The quantitation values are expressed as the relative fold change compared with the 16M CON group. The data are presented as the mean ± standard deviation (SD) (MyoG and MyHC, *n* = 10 per group; Myf5 and MyoD, *n* = 4 per group). ^*∗*^*p* < 0.05; ^*∗∗*^*p* < 0.01; and ^*∗∗∗*^*p* < 0.001.

To confirm the muscle protein synthesis, we investigated that SFe supplementation affected the expression of myosin heavy chain (MyHC) in TA muscle. The mRNA expression of myosin heavy chain I (MyHC 1) was lower in the 20M CON group than in the 16M CON group, but this decrease was inhibited by SFe supplementation in the 20M SFe group. The mRNA expressions of myosin heavy chain 2A (MyHC 2A) and myosin heavy chain 2X (MyHC 2X) were not significantly different among groups. The mRNA expression of myosin heavy chain 2B was higher in the 20M CON than in the 16M CON group but increased again in the 20M SFe group. When the mRNA expression of myogenic marker genes were evaluated in TA muscle, we found that the mRNA expression of MRF4 and myogenin (MyoG) was lower in the 20M CON group than in the 16M CON group, but this decrease was alleviated in the 20M SFe group (Figure 5(a)). In addition, the expression of MyoD, Myf5, MyoG, and embryonic-MyHC protein was also higher in the 20M SFe group than in the 20M CON group (Figure 5(b)). These data indicated that supplementation with SFe might increase the MyHC mRNA and protein expression through enhance myogenesis.

### 3.6. Supplementation with SFe Decreased mRNA and Protein Expression of Muscle Degradation Factors in Aged Mice

**Figure 6:**
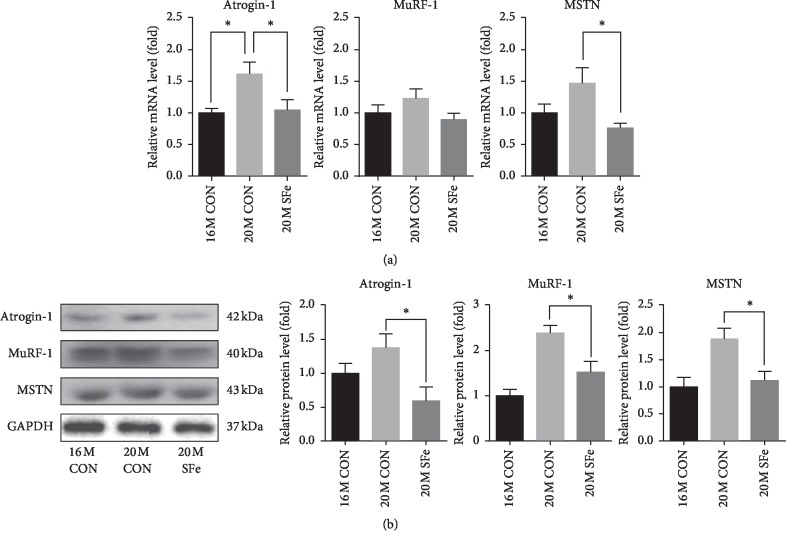
Supplementation with SFe decreased mRNA and protein expression of muscle degradation factors in aged mice. (a) The mRNA expression of atrogin-1, MuRF-1, and myostatin in TA muscle was analyzed by qRT-PCR, with 18S rRNA used as an internal control. The values are expressed as the relative fold change compared with the 16M CON group (*n* = 5–7 per group). (b) Atrogin-1, MuRF-1, and MSTN protein expression in TA muscle was analyzed by using western blotting, with GAPDH expression used as an internal control. Representative images of the blots are shown in the left panel; quantification of the blots was computed using ImageJ software and is shown in the right panel. The quantitation values are expressed as the relative fold change compared with the 16M CON group. The data are presented as the mean ± standard deviation (SD) (*n* = 10 per group). ^*∗*^*p* < 0.05.

To investigate whether SFe supplementation affected muscle protein degradation, we evaluated the expression of genes involved in muscle degradation, including myostatin (MSTN), MuRF-1, and atrogin-1, in TA muscle. As shown in Figure 6(a), the mRNA expression of atrogin-1 and MSTN expression levels was higher in the 20M CON group than in the 16M CON group, but this increase in expression was significantly lower in the 20M SFe group than in the 20M CON group. The mRNA expression of MuRF-1 mRNA appeared to be lower in the 20M SFe group than in the 20M CON group, but the difference was not significant. The protein expression in TA muscle was analyzed by western blotting and revealed that the expression of atrogin-1, MuRF-1, and MSTN was significantly lower in the 20M SFe group than in the 20M CON group (Figure 6(b)).

## 4. Discussion

As a result of the advances in medicine, the overall life expectancy has increased dramatically in the past century. According to the US Census Bureau, it is expected that the population over 65 years of age will rise from 319 million in 2014 to 417 million in 2060 [25]. In addition to regular physical activity and nutritional intervention, pharmacological approaches are needed to delay aging and prevent age-related diseases. In this study, we investigated the effects of SFe on the aging-associated decrease of insulin sensitivity and muscle function in a naturally aged mouse model. Our data showed that supplementation with SFe prevented the age-related increase of insulin resistance and improved glucose tolerance during aging. In addition, SFe supplementation prevented the increased fat mass and muscle loss that usually accompany aging. As skeletal muscle is the predominant tissue responsible for insulin-mediated glucose uptake [26], the increase of muscle mass might be a contributory factor to the improved insulin sensitivity.

Both dysregulation of muscle mass and muscle dysfunction are common in elderly people [27, 28]. Therefore, the maintenance of adequate muscle mass and function is important for the continuation of quality of life and the prevention of frailty. Our data showed that supplementation with SFe improved the grip strength of mice (Figure 3(a)). As it was reported that low grip strength was associated with the incidence of diabetes in middle-aged women [29], age-related muscle loss, known as sarcopenia, may result from insulin resistance in the muscle; thus, the improvements in insulin sensitivity after SFe supplementation may contribute to the increased muscle strength.

It was previously reported that denervation-induced muscle disuse causes atrophy of type 1 muscle fibers, causing slow-twitch fibers to change to fast-twitch fibers, decreasing type 1 fibers, and increasing type 2 fibers in the TA muscle of aged mice [6, 30]. Our results showed that the mRNA expression of MyHC1 was decreased by aging and increased by dietary supplementation with SFe (Figure 5(b)). In addition, PAS staining showed the number of type 1 muscles is significantly increased by SFe supplementation. These data indicated that the type of muscle fiber was changed from slow-twitch to fast-twitch fibers by aging; however, the type of muscle fiber was shifted from fast twitch to slow twitch by the dietary supplementation of SFe. Similar to our results, it was reported that the administration of SF extract in a rat model of disused muscle atrophy led to increased muscle mass in the gastrocnemius, TA, and soleus muscles and increased the shift in fiber type from fast-twitch to slow-twitch fibers [31].

Muscle protein metabolism is regulated by counterbalanced fluctuations in muscle protein synthesis and muscle protein degradation; however, this balance appears to be disturbed in the elderly and leads to age-induced muscle loss [32, 33]. It is known that Erk1/2 stimulates muscle cell differentiation and protein synthesis [23] and Erk1/2 phosphorylates the S6K1 [24]. Our results showed that both pErk1 and S6K1 were significantly increased in TA muscle of 20M SFe group than in the 20M CON group (Figure 4). Therefore, SFe supplementation had a beneficial effect of muscle mass through enhanced muscle protein synthesis.

In general, the muscle repair mechanism is mediated by satellite cells, which are muscle-specific stem cells. The satellite cells are quiescent in normal adult muscle and gradually differentiate to myoblasts, myocytes, and myofibers in a process called myogenic differentiation or myogenesis [34]. After satellite cell activation, some muscle regulatory factors, such as MyoD and Myf5 (myoblast marker genes) and myogenin and MRF4 (myocyte marker genes), are expressed [35, 36]. Our data indicated that dietary supplementation with SFe increased the expression of MyoD, Myf5, MyoG, and MRF4 myogenic factor (Figure 5(b)). In addition, SFe supplementation also increased multinucleated myofiber in aged mice (Figures 3(b) and 3(d)). Based on these results, we speculated that SFe might at least affect satellite cell activation. Further studies are required to elucidate whether SFe affects proliferation or activation of satellite cells or both. It was previously reported that the number of satellite cells were found in type 1 fiber higher than in type 2 fiber [37]. Satellite cell from slow-type-enriched soleus muscle generated slow-type myofibers [38]. Therefore, SFe supplementation exerted a protective effect against aging-induced muscle loss through the induction of muscle regeneration.

Insulin resistance can suppress the protein synthesis pathway and upregulate proteins in the degradation pathway, which consequently leads to muscle loss [39], as muscle mass is also regulated by degradation [40]. Muscle degradation is mediated by the activation of the ubiquitin-proteasome and the lysosomal pathway, including muscle-specific E3-ligases, atrogin-1, and MuRF-1 [41]. The secreted factor, myostatin, is an important negative regulator of skeletal muscle mass. MSTN is a member of the TGF-*β* protein family, which inhibits muscle differentiation and growth during myogenesis [42]. We found that dietary supplementation of SFe decreased the expression of MSTN, atrogin-1, and MuRF-1 in TA muscle. Our results were consistent with reports that SF decreased atrogin-1 and MuRF-1 gene expression in a human skeletal muscle cell line and in the muscles of a rat model of disuse-induced atrophy [16, 31].

Presently, we have not been able to identify the active component responsible for the observed beneficial effects on the muscles of aged mice. SF contains many bioactive compounds including lignans, triterpenoids, flavonoids, polyphenol, and polysaccharides [43], and lignans are known to be a major component of SF [43, 44]. Accordingly, we speculated that one or some lignans might be the active compound responsible for this effect, and further studies are required to identify the active ingredients.

Aging is related to a low-grade chronic inflammation [45]. Inflammation can increase the production of reactive oxygen species (ROS), which induce oxidative stress [46], and oxidative stress can also activate the inflammatory pathway [47], contributing to the development of age-related diseases. SF has various pharmacological effects, in particular, antioxidative and anti-inflammatory activities [11–15]. Therefore, these beneficial effects of SF may additionally act to improve insulin sensitivity and muscle function.

In conclusion, the dietary supplementation of SFe to aged mice improved insulin sensitivity, increased muscle mass and muscle strength through enhanced muscle regeneration, and reduced muscle degradation in aged mice. We suggest that SFe or components of SFe may be potential nutraceutical components for healthy aging.

## Figures and Tables

**Table 1:** Primer sets used for quantitative PCR analyses.

No.	Primer	Sense	Antisense
1	MuRF-1	AGGACTCCTGCAGAGTGACCAA	TTCTCGTCCAGGATGGCGTA
2	Atrogin-1	GCAAACACTGCCACATTCTCTC	CTTGAGGGGAAAGTGAGACG
3	Myostatin	GGCCATGATCTTGCTGTAAC	TTGGGTGCGATAATCCAGTC
4	18S rRNA	CCATCCAATCGGTAGTAGCG	GTAACCCGTTGAACCCCATT
5	MyoG	CCAACCCAGGAGATCATTTG	ACGATGGACGTAAGGGAGTG
6	MRF4	GGCCAAGTGTTTCGGATCATT	AAGAAAGGCGCTGAAGACTGC
7	MyHC 1	CCAAGGGCCTGAATGAGGAG	GCAAAGGCTCCAGGTCTGAG
8	MyHC 2A	AAGCGAAGAGTAAGGCTGTC	GTGATTGCTTGCAAAGGAAC
9	MyHC 2B	ACAAGCTGCGGGTGAAGAGC	CAGGACAGTGACAAAGAACG
10	MyHC 2X	CACCGTCTGGATGAGGCTGA	TGTTTGCGCAGACCCTTGATAG

## Data Availability

The data used to support the findings of this study are available from the corresponding author upon request.

## Abbreviations

Atrogin-1: Muscle atrophy F-box/atrogin-1
H&E: Hematoxylin and eosin
SF: *Schisandrae chinensis* Fructus
SFe: *Schisandrae chinensis* Fructus ethanol extract
S6K1: 70 kDa ribosomal protein S6 kinase 1
MSTN: Myostatin
MuRF-1: Muscle RING-finger protein-1
MyoG: Myogenin
MyHC: Myosin heavy chain
TA: Tibialis anterior.
